# Socioeconomic and contextual determinants of the burden of disease attributable to metabolic risks in childhood

**DOI:** 10.3389/fpubh.2022.1003737

**Published:** 2022-11-08

**Authors:** Laura Vallejo-Torres, Beatriz Gonzalez Lopez-Valcarcel

**Affiliations:** Department of Quantitative Methods in Economics and Management, University of Las Palmas de Gran Canaria, Las Palmas de Gran Canaria, Spain

**Keywords:** metabolic risks, burden of disease, childhood, inequalities, women empowerment

## Abstract

We analyze the socioeconomic and political contextual determinants of the burden of disease attributable to three metabolic risks in children: kidney dysfunction, high fasting plasma glucose, and high body-mass index. We use data from 121 countries. We matched data of the Global Burden of Disease project, World Bank and United Nations databases. The burden of disease is measured with the Disability Adjusted Life Years lost. We explore associations with four groups of variables: (i) income level, which measures differences in socioeconomic conditions between countries; (ii) income inequality, which measures within country inequalities in the income distribution; (iii) health care expenditure, which measures the resources allocated to health and healthcare, and (iv) women empowerment, which we measure in terms of both educational and political participation. Our findings point toward the need to act at the root of the underlying factors underpinning the disease burden, namely: reducing between and, particularly, within-country income inequalities, increasing the role of expenditure on health, and ensuring women empowerment and girls education. To our knowledge, this is the first study that have identified the associations of these variables with the burden of disease that is specifically attributable to metabolic risks in childhood.

## Introduction

Improving childhood health is a key goal worldwide and essential to promote and enhance adult health. While children mortality rates have decreased dramatically over the last decades (1), the rise in the prevalence among children of several risk factors with long-term consequences is a major concern. For instance, the prevalence of high systolic blood pressure (SBP) in childhood is substantial in many worldwide populations (2), and have been associated with high SBP in adulthood and with several other intermediate markers and hard outcomes of cardiovascular disease (CVD) later in life (3).

Most of these risk factors with growing prevalence during childhood play a large role in explaining overall population ill health. The Global Burden of Disease (GBD) project estimates that globally in 2019 61.9% (95% IC 59.8–63.9%) of the deaths and 48.1% (95% CI 45.3–50.1%) of the Disability-Adjusted Life Years (DALYs) were attributable to known risk factors (4). Across all ages, high SBP was the leading risk factor for attributable deaths, accounting for 19.2% of all deaths globally. In the general framework developed by the GBD project, risk factors are divided into three main categories that consist of environmental/occupational risks, behavioral risks, and metabolic risks. Behavioral risks are responsible of the largest share of attributable DALYs, both for the general population and among children (32.8 and 48.2%, respectively), followed by metabolic risks in the general population (18.3%), and by environmental/occupational risks in the children group (20.1%). Metabolic risk factors play a relatively small role in the attributable DALYs in childhood. In children aged 14 years and younger, only 0.4% of attributable DALYs are associated with metabolic risks (0.3, 1.1, and 1.2% for age groups 0–5, 6–9, and 10–14 year-old, respectively).

Notwithstanding the minor apparent role that metabolic risks play in children disease burden, the overwhelming consequences on mortality and morbidity associated with these risk factors in the long-run, and the significantly larger share that these metabolic risks play in the adult disease burden, highlight the need to act very early in life to reduce this burden. Exploring the complex socioeconomic and contextual patterns that emerge in the burden of disease attributable to metabolic risk factors in childhood provides a step forward in this direction, by helping to identify the roots in which to act to prevent the development and alleviate the consequences of exposure to such factors.

A number of socioeconomic and political contextual variables have been found to be linked with several population health outcomes and, particularly, with children health. Both income and income inequalities are main drivers of variations in population health. Primarily, socioeconomic conditions are considered a structural determinant of health, responsible for a major part of health inequities observed between and within countries (5, 6). Furthermore, within country income inequalities play an additional role in harming population health, both directly by increasing poverty (the absolute income effect) and causing chronic stress due to social comparisons (the relative income effect), and indirectly by eroding societal trust and destabilizing communities (the contextual effect) (7). With respect to health expenditure, effective health care services are required to improve the health of the individuals, and while establishing a causal link between health spending and health outcomes is a complex task (8), several studies have found a positive impact of health spending on several population and children health outcomes (9–11). Finally, the impact of women empowerment on child health is the focus of a growing body of literature. Improving women's education and literacy not only has a direct effect on their own health and well-being, but also on their abilities and resources required to provide appropriate caregiving for children, a role mostly fulfilled by women (12, 13). Moreover, women political empowerment not only has an “instrumental role” in making their voices more influential and in promoting policies designed to respond to their needs and concerns, but also it plays a “constructive role” in contributing to shape society's values and priorities, which have been in turn associated with improved women's well-being and child health outcomes (14, 15).

The aim of this study is to explore the association of these variables with the burden of disease that is specifically attributable to metabolic risk factors in childhood. We measure the income and income inequality gradient and the associations between health spending and women empowerment with the burden associated with the three metabolic risk factors in childhood measured at the GDB project, namely: kidney dysfunction, high fasting plasma glucose (FPG), and high body-mass index (BMI)^1^.

## Materials and methods

In this paper we analyze the socioeconomic and political contextual determinants of the burden of disease attributable to metabolic risks in children using data from 121 countries. We explore the impact of four groups of variables: (i) income level, which measures differences in socioeconomic conditions between countries; (ii) income inequality, which measures within country inequalities in the income distribution; (iii) health care expenditure, which measures the resources allocated to health and healthcare, and (iv) women empowerment, which we measure in terms of both educational and political participation. This is an observational ecological retrospective study for 121 countries.

We compiled information from three sources of data: (i) the GBD project database^2^ for information on the rate of DALYs (measured by DALYs per 100,000 population) attributed to each metabolic risk in children, (ii) the World Bank database^3^ for information on income levels and income inequality indices, and (iii) the United Union database^4^ for information on health spending and women empowerment measures. Databases were linked by using a unique country-specific identifier. We used data from 2019 and from 121 countries; these were countries with information available on the four sets of independent variables under study (see list of countries in Appendix 1). When information for the year 2019 was missing we used data from the nearest available year.

We analyzed three dependent variables that measure the rate of DALYs in children attributable to kidney dysfunction, high FPG and high BMI, respectively. For each variable, we conducted separate linear regression models by age intervals: 0 to 5, 6 to 9, and 10 to 14 year-old. Independent variables included: (i) income group as defined by the World Bank classification into low, lower-middle, upper-middle, and high-income countries; (ii) income inequality, measured by the Gini index which ranges from 0 to 100, where 0 represent perfect equality and 100 represents perfect inequality; we created a binary variable indicating whether the country belongs to the most unequal quartile (i.e., Gini index higher than 42); (iii) resources allocated to health and healthcare, measured by health expenditure as a share of total government expenditure; and (iv) women empowerment, measured by the ratio girls/boys in primary, secondary, and university education and by the percentage of seats held by women in national parliaments. Table 1 provides the definition of all variables and their sources.

**Table 1:** Variable definitions and sources.

**Variables**		**Definition**	**Source**
Dependent variables	DALYs attributable to high body-mass index (rate per 100,000 population)	Disaggregated in three age intervals: 0–5 years 6–9 years 10–14 years	GBD 2019
	DALYs attributable to fast plasma glucose (rate per 100,000 population)		GBD 2019
	DALYs attributable to kidney dysfunction (rate per 100,000 population)		GBD 2019
Economic variables: income and inequality in income	Group of per capita income	Low income (reference) Lower-middle income Upper-middle income High income	World Bank
	Inequality in income distribution	Binary variable taking value = 1 if country belongs to the most unequal quartile; = 0 otherwise	Word Bank
Resources allocated to health and healthcare	Health expenditure	Domestic general government health expenditure (% of total government expenditure)	United Nations
Women education and political empowerment	Primary education	Ratio girls/boys in primary education	United Nations
	Secondary education	Ratio girls/boys in secondary education	United Nations
	University education	Ratio girls/boys in university education	United Nations
	Women in national parliament	Seats held by women in national parliament (%)	United Nations

We conducted multivariate ordinary least squared (OLS) models and reported standardized coefficients, which allow the comparison of the relative importance of each independent variable in explaining the dependent variables. Coefficients with associated *p*-values equal or under 0.05 were considered strongly significant, while *p*-values equal or under 0.10 were considered weakly significant.

We checked the assumptions of the linear regression model. Multicollinearity among independent variables was explored with the Variance Inflation Factor (VIF), with values higher than 10 indicating a multicollinearity problem (16). Ramsey Reset test (17) was used to test for specification errors and, specifically, to test for non-linearities among the dependent and the independent variables. When non-linearities were identified, we tested for the inclusion of quadratic functions of continuous independent variables. Quadratic forms were kept in models when they were found to be statistically significant and when their inclusion solved misspecification issues. White test was used for heteroskedasticity as a general asymptotic test when the functional form of the random error's variance is unknown (18). We calculated and reported robust standard errors of the regression coefficients to avoid the estimation bias in case of heteroskedasticity. Shapiro-Wilk test was used for testing normality of errors (19). Analyses were undertaken using Stata version 17 (20).

## Results

Table 2 presents full model results for each dependent variable and for each age group separately. Reduced models that only kept significant variables yield the same conclusions (results not shown but available from the authors upon request).

**Table 2:** Model results of the socioeconomic and contextual determinants of DALYs attributable to metabolic risk factors in children.

	**Kidney dysfunction** **Stand. coeff**. **(*****p*****-value)**			**High FPG** **Stand. coeff**. **(*****p*****-value)**			**High BMI (obesity)** **Stand. coeff**. **(*****p*****-value)**		
	**Age group** **0 to 5**	**Age group** **6 to 9**	**Age group** **10 to 14**	**Age group** **0 to 5**	**Age group** **6 to 9**	**Age group** **10 to 14**	**Age group** **0 to 5**	**Age group** **6 to 9**	**Age group** **10 to 14**
**Income level**	low income–omitted category			low income–omitted category			low income–omitted category		
Lower-medium income	**–**0.294 (0.143)	−0.044 (0.710)	−0.019 (0.855)	−0.317* *(0.071)*	0.076 (0.377)	0.137 (0.251)	−0.202 (0.296)	−0.045 (0.692)	−0.004 (0.966)
Upper-medium income	−0.482** **(0.021)**	−0.279** **(0.018)**	−0.261** **(0.022)**	−0.483** **(0.009)**	−0.155* *(0.089)*	−0.082 (0.518)	−0.031 (0.891)	0.209 (0.156)	0.174 (0.140)
High income	−0.661*** **(0.001)**	−0.562*** **(0.000)**	−0.598*** **(0.000)**	−0.596*** **(0.001)**	−0.221** **(0.016)**	−0.129 (0.313)	−0.180 (0.449)	0.240 (0.143)	0.400** **(0.005)**
**Income inequality** (most unequal quartile)	0.144 (0.134)	0.161** **(0.016)**	0.180** **(0.008)**	0.143* *(0.070)*	0.074 (0.246)	0.161* *(0.100)*	0.329** **(0.003)**	0.246** **(0.010)**	0.198** **(0.020)**
**Health expenditure** (% of public expenditure)	−0.052 (0.457)	−0.116 (0.135)	−0.086 (0.292)	−0.153 (0.127)	−0.233** **(0.026)**	−0.228** **(0.018)**	−0.561 (0.216)	−0.695 (0.109)	−0.787** **(0.039)**
Health expenditure squared (% of public expenditure)	NA	NA	NA	NA	NA	NA	0.795* *(0.052)*	1.045** **(0.024)**	1.187** **(0.004)**
**Women empowerment**									
Primary education Girls/Boys	−0.147 (0.104)	−0.116* *(0.205)*	−0.151** **(0.034)**	−0.131* *(0.100)*	−0.075 (0.573)	−0.054 (0.624)	−0.005 (0.955)	0.090 (0.138)	0.073 (0.208)
Secondary education Girls/boys	−0.043 (0.706)	−0.074 (0.409)	−0.086 (0.249)	0.172* *(0.094)*	0.136 (0.225)	0.128 (0.328)	0.094 (0.455)	0.058 (0.538)	−0.039 (0.640)
University education Girls/boys	−0.132 (0.287)	−0.106 (0.244)	−0.066 (0.396)	−0.252 (0.284)	−0.218 (0.121)	−0.201 (0.119)	−0.101 (0.477)	−0.085 (0.439)	−0.141 (0.186)
Women in parliament	*−0.114* *(0.122)*	−0.115** **(0.046** ***)***	−0.117** **(0.045)**	0.037 (0.646)	0.023 (0.626)	−0.003 (0.969)	0.104 (0.352)	0.063 (0.425)	0.063 (0.351)
R square	0.586	0.6531	0.7006	0.5037	0.348	0.3121	0.2919	0.4074	0.4639
Ramsey RESET test	0.610	0.565	0.170	2.100	2.84**	3.57**	1.350	1.410	2.050
White test heteroskedasticity	95.29***	63.14*	61.31*	81.06***	61.15*	60.10*	59.55	75.46*	76.76**
Shapiro-Wilk test normality	4.064***	3.311***	3.560***	4.105***	4.202***	3.240***	5.612***	4.917***	4.991***
Sample size	121	121	121	121	121	121	121	121	121

Stand. coeff., Standarised coefficients; NA, Non Applicable. ***p < 0.001; **p < 0.05; *p < 0.1. Coefficients in bold are strongly significant and in italic weakly significant. P-values are based on robust standard errors.

We found no evidence of multicollinearity issues. The maximum VIF in the linear models is 5.60 (for high income countries), and the mean VIF is 2.66. Linear models for all age groups on kidney dysfunction and on FPG for the youngest age group had no evidence of misspecification errors. In contrast, models for obesity passed the Ramsey Reset test only after the inclusion of health expenditure in quadratic form. We could not identify well-specified models on FPG for the older age groups at a 5% significance level, however, the null hypothesis that the linear models were well-specified could not be rejected at a 1% significance level. We found evidence of heteroskedasticity in nearly every model, and therefore we reported robust standard errors in every case. The assumption of nonnormality of errors was rejected in every model, but after the results of misspecification tests, we considered the model inference as asymptotically valid (16). Given the sample size of our models we do not need to rely on small sample properties but on the asymptotic properties of estimates.

We observed a strong and significant income gradient in the burden of kidney dysfunction among children in every age group. The income category variables had the largest relative importance in assessing differences in the disease burden attributable to this metabolic risk across countries, followed by the income inequality variable. This latter variable was significant in models that focused on the burden among 6 to 9 and 10 to 14 years-old children, and suggest that countries with the highest levels of income inequality experience a larger burden of disease attributable to kidney dysfunction in these age groups, even after controlling for the absolute income level. The share in health spending had not a significant association with the burden due to kidney dysfunction in children, but some indicators of women empowerment were identified as negatively associated, either weakly or strongly significantly, with the burden of kidney dysfunction in every age group; particularly, the ratio girls/boys in primary education and the percentage of women holding seats at the country parliament.

In the case of the burden due to high FPG in childhood, we observed a strong and significant income gradient among the youngest children groups. Income inequality had a weakly significant association in the youngest and oldest age groups, and also pointed toward the most unequal countries suffering a higher disease burden associated with high FPG. In addition, the models showed that a higher share of total public expenditure allocated on health and health care was associated with lower burden of FPG among children. This variable was found to be strongly significant at the two oldest age groups. Some indicators of women participation in education were weakly associated with the burden attributable to high FPG among the youngest children, which in the case of FPG were related to the ratio girls/boys in primary and secondary education.

**Figure 1:**
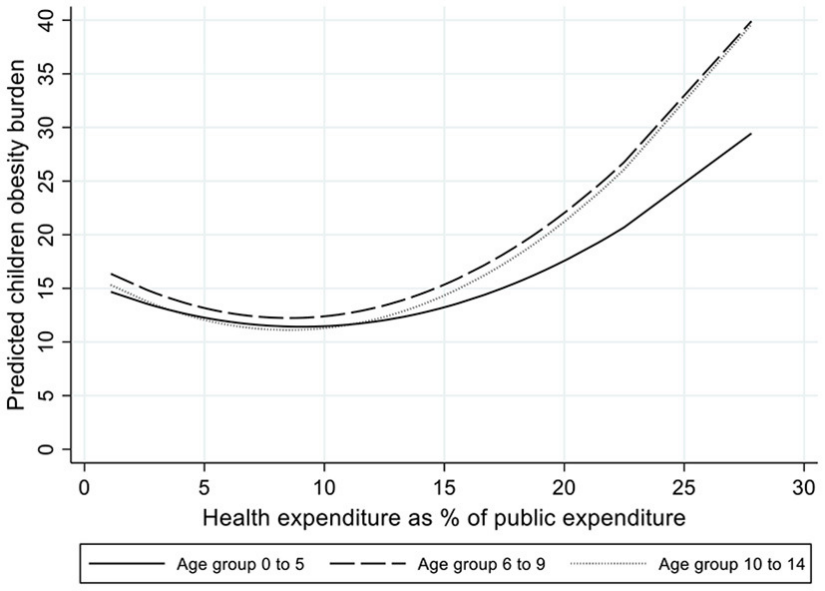
Predicted children obesity burden by level of health expenditure as percentage of public expenditure.

The socioeconomic and contextual determinants of the burden attributable to obesity in childhood showed some different patterns. The income gradient was either non-present or had a reverse relationship, indicating that in high income countries the burden associated with high BMI is larger than in other countries for the oldest children. Income inequality has a positive and significant effect at every age group, which suggest that, similarly to that observed in the other risk factors, independently of the country level of income, the most unequal countries experience a higher disease burden attributable to children obesity. However, the association of health spending with obesity was found to be non-linear, showing that at low levels of health spending, increases in this variable were associated with decreases in the obesity burden, while at higher levels of health spending the association turned positive (see Figure 1). We will discuss this result in the discussion section. According to our models, women empowerment indicators did not appear to be associated with children obesity burden.

## Discussion

In this paper we have analyzed the determinants of disease burden attributable to metabolic risk factors in childhood. Our results showed complex and varying socioeconomic and contextual patterns. We observed that while kidney dysfunction and high FPG had a strong income gradient-indicating that poorer countries experienced a disproportionately higher burden associated with these metabolic risks, the opposite was true for the burden attributable to high BMI among older children. However, the finding that income inequality was positively associated with disease burden was consistent across the three analyzed risk factors, albeit of being only weakly significant for high FPG. Higher shares of public spending allocated to health were found to be negatively associated with the burden of disease attributable to high FPG, while the relationship with the obesity burden was found to be non-linear. Women empowerment indicators were found to be negatively correlated with the disease burden attributable to high FPG and kidney dysfunction.

Among the three metabolic factors analyzed in this study, obesity is mostly a consequence of behavioral risks, such as unhealthy diets and sedentarism, which mainly characterized richer economies [although some low- and middle- income countries are showing rapid rises in children obesity, despite continuing high levels of undernutrition (21)]. On the other hand, the development of kidney dysfunction and high FPG in childhood are mainly due to congenital factors, but their consequences are, nonetheless, partly amenable to health care by early detection and appropriate treatment. This might, to some extent, explain the differences in the income gradient and in the role of health care spending observed in the analyses conducted across these risk factors. The fact that the association of children obesity with the share of public spending allocated to health was found to be non-linear might suggest that, across countries with low health spending, increases in health expenditure are associated with decreases in children obesity, but that among countries with larger proportions of spending on health care, further increases are associated with increases in the obesity burden. Due to the ecological nature of the data used in this study, it is difficult to conclude whether this result implies an ecological fallacy (i.e., the findings associated with aggregated country-level data do not reflect the situation of the individuals belonging to these countries). Alternatively, this result might suggest that, after achieving a certain level of health spending, there are other areas of public spending that are more effective and cost-effective at reducing the obesity burden. Rather than pharmacological or surgical interventions, the most effective and cost-effective strategies to prevent and reduce children obesity concern other public policies on housing, urban environment, transportation, or actions to reduce poverty, and they involve multiple strategies that focus on meals, classroom activities, sports, and play activities, and involve home, school or kindergarten, and community participants (21).

The strong and consistent finding that the most unequal the country the higher the burden of disease associated with each metabolic risk analyzed in this study is striking. It highlights how, over and above the absolute income level of a country, within-country inequalities are main drivers of disease burden in childhood. This finding emphasizes the need to prioritize efforts at reducing income inequalities, on top of ensuring economic growth, in order to reduce the development and consequences of metabolic risks factors in children. Finally, the findings that indicators of women empowerment might act as protective factors for the burden of some of the risk factors analyzed in this study is also crucial. This result supports the claim that, aside from its own value, empowering women may improve women's own health and also that of the next generation (15). The absence of an effect of women empowerment on the burden associated to high BMI could, to some extent, be explained by the controversial argument that rises in women employment are partly responsible of the increases in children and adolescence obesity. However, the empirical literature in this area has found conflicting results (22–34), and our finding simply suggest that women empowerment does no harm nor good at reducing children obesity burden.

This study has a number of limitations. First, we have analyzed three metabolic risk factors which are measured to be directly attributable to the burden of disease in children. However, other key metabolic risk factors include high LDL cholesterol, high SBP and low bone density mineral. While these three metabolic risk factors are not directly attributed to deaths and DALYs in childhood, they play an indirect effect due to the interrelated associations between them and other risk factors. For instance, obesity is associated with high SBP, high FPG and high LDL cholesterol, and high SBP is both a cause and a consequence of kidney dysfunction. Therefore, there are several interrelationships between these metabolic risk factors. In addition, we have used data for 2019 and from 121 countries. Establishing causal relationships using cross-sectional ecological data is challenging for two main reasons. First, our independent variables might be correlated with a number of factors that also affect children disease burden, and this might bias our estimates due to unobserved country-level heterogeneity. And secondly, the ecological nature of the data implies that attention is needed when trying to generalize findings based on comparisons across countries to the population within the countries. Nonetheless, we have been able to identify strong associations between several socioeconomic and contextual factors and the burden attributable to metabolic risk factors in childhood.

In summary, in this study we have investigated the determinants of the disease burden attributable to metabolic risk factors in children, and we have found several socioeconomic and contextual patterns. Our findings point toward the need to act at the root of the underlying factors underpinning the disease burden, namely: reducing between and, particularly, within-country income inequalities, increasing the role of expenditure on health, but also that of other public measures, and ensuring women empowerment and girls education. To our knowledge, this is the first study that have identified the association of these variables with the burden of disease that is specifically attributable to metabolic risks in childhood. Given the large role that metabolic risks play at the population disease burden, reducing the onset of and alleviating the consequences of metabolic risk factors early in life will significantly reduce the overall population disease burden.

## Data availability statement

Publicly available datasets were analyzed in this study. This data can be found at: the Global Burden of Disease repository (available from: https://www.healthdata.org/results/gbd_summaries/2019), the World Bank repository (available from: https://data.worldbank.org/), and the United Union repository (available from: http://data.un.org/).

## Author contributions

LV-T and BG contributed to the design, data analysis, interpretation of results, and drafting the manuscript. Both authors contributed to the article and approved the submitted version.

## Funding

This publication is based on the work of the COST Action HyperChildNET (CA19115), with the support of COST (European Cooperation in Science and Technology) and the Horizon 2020 Framework Program of the European Union.

## Conflict of interest

The authors declare that the research was conducted in the absence of any commercial or financial relationships that could be construed as a potential conflict of interest.

## Publisher's note

All claims expressed in this article are solely those of the authors and do not necessarily represent those of their affiliated organizations, or those of the publisher, the editors and the reviewers. Any product that may be evaluated in this article, or claim that may be made by its manufacturer, is not guaranteed or endorsed by the publisher.

## Footnotes

^1^The metabolic risks included in the GBD data consist of a total of six categories: high LDL cholesterol, high SBP, low bone density mineral, high fasting plasma glucose (FPG), high body-mass index (BMI) and kidney dysfunction. Of these, only the last three factors are estimated to be direct risk factors for the burden of disease in children.

^2^Available from: https://www.healthdata.org/results/gbd_summaries/2019.

^3^Available from: https://data.worldbank.org/.

^4^Available from: http://data.un.org/.

## Appendix

List of included countries.

**Table:**

Albania	Georgia	North Macedonia
Angola	Germany	Norway
Argentina	Ghana	Pakistan
Armenia	Greece	Panama
Australia	Guatemala	Paraguay
Austria	Guinea	Peru
Bangladesh	Honduras	Philippines
Belarus	Hungary	Poland
Belgium	Iceland	Portugal
Benin	Indonesia	Romania
Bhutan	Iran, Islamic Rep.	Russian Federation
Botswana	Ireland	Rwanda
Brazil	Israel	Senegal
Bulgaria	Italy	Serbia
Burkina Faso	Kazakhstan	Seychelles
Burundi	Kenya	Slovak Republic
Cabo Verde	Korea, Rep.	Slovenia
Cameroon	Kyrgyz Republic	South Africa
Canada	Lao PDR	Spain
Chad	Latvia	Sri Lanka
Chile	Lesotho	Sudan
China	Liberia	Sweden
Colombia	Lithuania	Switzerland
Comoros	Luxembourg	São Tomé and Principe
Costa Rica	Malawi	Tajikistan
Croatia	Malaysia	Tanzania
Cyprus	Maldives	Thailand
Czech Republic	Malta	Timor-Leste
Côte d'Ivoire	Marshall Islands	Togo
Denmark	Mauritania	Tunisia
Djibouti	Mauritius	Turkey
Dominican Republic	Mexico	Ukraine
Ecuador	Mongolia	United Arab Emirates
Egypt, Arab Rep.	Montenegro	United Kingdom
El Salvador	Morocco	United States
Estonia	Mozambique	Uruguay
Eswatini	Myanmar	Zimbabwe
Ethiopia	Namibia	
Finland	Netherlands
France	Nicaragua	
French Polynesia	Niger	
Gambia, The	Nigeria	
